# Supplementary material for: The Response to Inoculation with PGPR Plus Orange Peel Amendment on Soybean Is Cultivar and Environment Dependent
Source: Plants (Basel). 2022 Apr 22;11(9):1138. doi: 10.3390/plants11091138 (PMC9104577; doi:10.3390/plants11091138)
Supplement: Supplementary file 1 [file plants-11-01138-s001.zip › plants-1679015-supplementary.pdf]

Supplementary Table S1. Characteristics of the soybean cultivars tested in the preliminary greenhouse experiment.

Cultivar	Brand/Company	Maturity Group	Trait
AG44X0	Asgrow	4.4	R2X[1]
AG53X0	Asgrow	5.3	R2X
AG69X0	Asgrow	6.9	R2X
CZ 4539GTLL	Credenz	4.5	GTLL[2]
CZ 5859LL	Credenz	5.8	LL[3]
CZ 6515LL	Credenz	6.5	LL
G4190RX	Agrigold	4.1	R2X, STS[4]
G5000RX	Agrigold	5.0	R2X, STS
GoSoy 512E18	Stratton Seed Co.	5.1	Enlist[5]
LS4798X	Local Seed Co.	4.7	R2X
LS5087X	Local Seed Co.	5.0	R2X
LS5588X	Local Seed Co.	5.5	R2X
LSX6501XS	Local Seed Co.	6.5	R2X, STS
S49-F5X	NK Seeds	4.9	R2X
REV 4940X	REV Brand Seeds	4.9	R2X
REV 5659X	REV Brand Seeds	5.6	R2X
S49XT39	Dyna-Gro	4.9	R2X
S52XT08	Dyna-Gro	5.2	R2X
S54XT17	Dyna-Gro	5.4	R2X
S56XT99	Dyna-Gro	5.6	R2X

[1]Roundup Ready 2 Xtend® (R2X)
[2]LibertLink® GT27® (GTLL)
[3]LibertLink® (LL)
[4]Sulfonylurea Tolerant Soybean (STS®)
[5]Enlist E3® (Enlist)

Supplementary Table S2. Author, publication year, soybean shoot B values (used to calculate the proportion of nitrogen derived from the air), and maturity stage retrieved from literature for soybean grown in the greenhouse experiments.

Author	Publication year	Shoot B value %	Stage
Amarger	1979	-1.2	R4-R5
Amarger	1979	-1.5	R4-R5
Amarger	1979	-1.6	R4-R5
Amarger	1979	-1.3	R4-R5
Bergersen et al.	1985	-1.36	R3-R5
Bergersen et al.	1985	-1.3	R4-R5
Bergersen et al.	1985	-1.4	R4-R5
Schipanski et al.	2010	-2.28	R4
Schipanski et al.	2010	-2.7	R4
Balboa & Ciampitti	2020	-2.37	R3
Balboa & Ciampitti	2020	-2.32	R5
Balboa & Ciampitti	2020	-2.05	R3
Balboa & Ciampitti	2020	-1.97	R5
Balboa & Ciampitti	2020	-1.96	R3
Balboa & Ciampitti	2020	-2	R5
Balboa & Ciampitti	2020	-2.18	R3
Balboa & Ciampitti	2020	-2.2	R5
**Average**		**-1.86**	

Supplementary Table S3. Author, publication year, soybean shoot B values (used to calculate the proportion of nitrogen derived from the air), and maturity stage retrieved from literature for soybean cultivated in field conditions.

Author	Publication year	Shoot B value %	Stage
Araujo et al.	2018	-2.85	R1
Araujo et al.	2018	-3.17	R1
Araujo et al.	2018	-2.76	R1
Araujo et al.	2018	-2.35	R2
**Average**		**-2.78**	
